# Physiological changes and transcript identification in *Coreopsis tinctoria* Nutt. in early stages of salt stress

**DOI:** 10.7717/peerj.11888

**Published:** 2021-08-09

**Authors:** Hong Jiang, Zhiyuan Li, Xiumei Jiang, Yong Qin

**Affiliations:** College of Forestry and Horticulture, Xinjiang Agriculture University, Urumqi, China

**Keywords:** *Coreopsis tinctoria* Nutt., Salt stress, Physiological, Transcriptome

## Abstract

*Coreopsis tinctoria* Nutt. (*C. tinctoria*) is a special tea ingredient that adapts to certain salt stresses and shares the functions of chrysanthemum. With annual expansion of the cultivation area of *C. tinctoria* in Xinjiang (China), soil salinity may become a constraint for chrysanthemum cultivation. To investigate the response of C. tinctoria to salt stress, physiological and transcriptional changes in *C. tinctoria* in the early stages of low (50 mM NaCl) and high (200 mM NaCl) salt stress were analyzed and identified. The results showed that the contents of osmotic regulators (free proline, soluble sugar, and soluble protein) and antioxidant enzymes (catalase and peroxidase) under salt stress increased to various extents compared with those of the control (CK) within 72 h, and the increase was higher under 200 mM NaCl treatments. De novo RNA-seq was used to analyze changes in the transcripts under 50 and 200 mM NaCl treatments for up to 48 h. In total, 8,584, 3,760, 7,833, 19,341, 13,233, and 9,224 differentially expressed genes (DEGs) were detected under 12 h, 24 h, and 48 h for 50 and 200 mM NaCl treatments, respectively. Weighted correlation network analysis (WGCNA) was used to analyze the correlations between all DEGs and physiological indexes. We found that the coexpression modules blue2 and Lightskyblue4 highly correlated with osmotic regulators and CAT and identified 20 and 30 hub genes, respectively. The results provide useful data for the further study of salt tolerance in *C. tinctoria*.

## Introduction

Increased salt in soil affects plant growth and development by causing osmotic stress, ion toxicity and oxidative stress. Plants generally adapt to a saline environment via the accumulation of osmotic regulatory substances, metabolites, and antioxidant enzymes and regulation of signal transduction factors and salt stress-related genes. A large number of studies showed that various physiological indexes of plants, such as free proline (Pro), soluble sugar, soluble proteins, and antioxidant enzymes, undergo different levels of response under different salinity levels and that different plant species, such as *Carthamus tinctorius* L. (Han et al., 2014), *Apocynum venetum* L. (Ning et al., 2010), *Lycium ruthenicum* Murr. (Li et al., 2019), and *Salicornia europaea* L. (Liu et al., 2013), synthesize osmotic regulatory substances to improve the activity of antioxidant enzymes. Some studies on *C. tinctoria* also indicated that soluble sugar, soluble protein, and Pro contents increased with increases in different types of salinity (NaCl, Na_2_SO_4_, and mixed salt), and the activity of superoxide dismutase (SOD) and peroxidase (POD) increased initially then decreased, which indicated that *C. tinctoria* could adapt to a certain salinity (NaCl) through adjustment of physiological mechanisms (Yeergen-Xiyimulati, 2015). Some key biological processes and related genes are activated to play a role in plant responses to stress. RNA-seq was widely used to study abiotic stress responses in many plants. For example, 11 highly expressed transcription factor genes (e.g., *HD-ZIP, ERF,* and *G2-like*) were screened from different genotypes of upland cotton (Peng et al., 2017). The *GmIFS1* gene in transgenic soybean and tobacco was highly induced by salt and significantly increased the isoflavone content and enhanced salt tolerance (Jia et al., 2016). The products of some genes (*HPT, GGT, AP, 6-PGD,* and *G6PDH*) in *Lolium multiflorum* L. act as antioxidants in lipid metabolism and signal transduction pathways to improve drought resistance (Pan et al., 2016). Other studies showed that the maintenance of photosynthesis should improve the drought tolerance of rice (Ma et al., 2016). Strongly induced salt response genes in two different genotypes of sesame were mainly related to amino acid metabolism, carbohydrate metabolism, the biosynthesis of secondary metabolites, plant hormone signal transduction, and redox processes (Zhang et al., 2019). Plants showed different responses under different abiotic stresses. Analyses of plant transcriptome revealed many differentially expressed genes related to specific stresses, such as salt stress, temperature stress, water stress and drought stress. A clear understanding of the tolerance mechanism of related stresses can provide a theoretical basis for the study of the molecular mechanism of plant stress resistance and the cultivation of stress-resistant varieties.

*C*. *tinctoria* (*Coreopsis tinctoria* Nutt.) is a new and well-known functional chrysanthemum tea material in China. It is mainly produced in high-altitude areas such as the Kunlun Mountains in Xinjiang, China. *C. tinctoria* inhabits a hostile primitive environment and offers a limited yield as a material with important potential medicinal value (Wang et al., 2015). Modern pharmacology confirmed that it serves as an antioxidant; reduces blood pressure, sugar (Dias et al., 2010) and fat (Zhu et al., 2020); and exerts anticancer effects (Dias et al., 2012; Qiao et al., 2019; Yang, Peng & Lu, 2019). To meet market demand, people recently began cultivating it in many regions of the Xinjiang Plain (Yeergen-Xiyimulati, Qin & Xu, 2014). Saline-alkali soil is widely distributed in Xinjiang, where a great variety of salt types exist. As a result of excess irrigation, the area of secondary salinized soil has increased annually, and soil salinization has seriously constrained the yields of various types of crops (Lu et al., 2013). Soil salinization will become a factor restricting the development of *C. tinctoria*. Some scholars have conducted basic research on the salt tolerance of *C. tinctoria*. Guo et al. (2013) found that the suitable range of salt tolerance of *C. tinctoria* seeds was 0−1.3% and the limit concentration of salt tolerance was 2.23% (Yeergen-Xiyimulati, Qin & Xu, 2014). With increasing NaCl concentration, the plant height and biomass of *C. tinctoria* seedlings decreased, the soluble sugar, soluble protein, Pro and malondialdehyde (MDA) contents in the leaves increased, and the activities of SOD and POD first increased then decreased (Yeergen-Xiyimulati, 2015). These studies showed that the seeds and seedlings of *C. tinctoria* has a certain salt tolerance. However, research on salt stress in *C. tinctoria* is mainly focused on physiology, and few in-depth studies on molecular biology exist. Therefore, the stress resistance mechanism of *C. tinctoria* is not well understood.

To further understand the salt tolerance of *C. tinctoria*, two levels of salt stress (50 and 200 mM NaCl) were designed to study the early physiological changes of *C. tinctoria* after salt stress. *De novo* RNA-seq was used to analyze and identify the signaling pathways and candidate genes related to salt stress. The results of the present study provide valuable information on the response of *C. tinctoria* to low and high salt stress. These results provide a theoretical basis for the further study of salt tolerance mechanisms and the breeding of salt-tolerant varieties of *C. tinctoria* in the future.

## Materials & Methods

### Plant materials and treatment

*C. tinctoria* seeds were collected from Hotan, China (altitude, 2,196 m, N: 37°37′17.00″E: 78°16′58.80″). The seeds were planted in an artificial-lighting hydroponic plant factory under 10 h of artificial light (8,100 1×  light intensity, 25–28 °C), 14 h of darkness (20–22 °C), and a relative humidity of 60–70%. A total of 300 undamaged seeds of the same size were selected, sterilized and sown in Petri dishes. After the seeds germinated for 15 days (2 leaves), seedlings with consistent growth were transferred to a 6-L hydroponic container containing 1/2 Hoagland nutrient solution for continuous cultivation. During this period, the nutrient solution was changed every 5 days, and an oxygen pump was used to supply oxygen regularly for 6 h a day. On the 30th day (when the plants had 4–6 leaves), 0 (control (CK)), 50 (FS), and 200 (TS) mM NaCl solutions were used as salt stress treatments. Leaves of *C. tinctoria* were quickly cut off at 12 h, 24 h, and 48 h (the second pair of functional leaves from bottom to top) and put into liquid nitrogen at −80 °C for cold storage. For each time point, three biological repetitions were conducted, with 30 seedlings per repetition.

### Physiological index and growth determination

The MDA, Pro, soluble sugar and soluble protein contents were determined by the thiobarbituric acid (TBA) colorimetric method, indene triketone staining method, anthrone colorimetric method and Coomassie brilliant blue GMel 250 colorimetric method (Gao, 2006). Superoxide dismutase, POD, catalase (CAT), and ascorbate peroxidase (APX) were detected by Rayto RT-6100 enzyme colorimetry and an Elabscience plant kit (Wuhan, China).

The fresh weight and the root/shoot ratio of the plants were determined by the following method. The seedlings were removed from the nutrient solution, washed clean with deionized water, and quickly dried with absorbent paper. The above- and belowground fresh weights were measured, and the root/shoot ratio (underground fresh weight/aboveground fresh weight) was calculated (Rahimzadeh Karvansara & Razavi, 2019).

### RNA Extraction, Library Establishment and Sequencing

RNA extraction, library establishment and sequencing were performed similarly to previously reported methods (Xue et al., 2020). The total RNA of 21 leaf samples of *C. tinctoria* was extracted by the TRIzol (Invitrogen) method, and the quality of RNA samples was detected by agarose gel electrophoresis, a Qubit 2.0 Fluorometer (Life Technologies, CA, USA) and an Agilent 2100 Bioanalyzer (Agilent Technologies, CA, USA) to ensure that qualified samples were used for transcriptome sequencing.

The cDNA library was established by a TruSeq^*TM*^ RNA sample preparation kit (Illumina, San Diego, CA). mRNA with a polyA tail was enriched with oligo(dT) magnetic beads, and the mRNA was broken into short fragments with the addition of fragmentation buffer. Using the short fragment mRNA as the template, first-strand cDNA was synthesized by six-base random primers (random hexamers). Buffer, dNTPs (dUTP, dATP, dGTP and dCTP) and DNA polymerase I were then added to synthesize double-stranded cDNA. The double-stranded cDNA was then purified by AMPure XP beads. The double-stranded cDNA structure had a sticky end that was filled until blunt via the addition of End Repair Mix, and an A base was added at the 3′ end to overlap with a T base overhang. The fragment size was then selected by AMPure XP beads, and the final cDNA library was enriched by PCR as described in the instructions. After cDNA enrichment by PCR, a Qubit 2.0 instrument was used for preliminary quantification, and an Agilent 2100 instrument was used to detect the insert size of the library. The effective concentration of the library was subsequently determined by Q-PCR (effective concentration of the library > 2 nM), and the Illumina HiSeq platform was used for sequencing (Wuhan Metware).

### Data filtering and reassembly of the transcriptome

The raw data of 21 samples were obtained by *de novo* transcriptome sequencing, and the raw data underwent filtering and quality control before reassembly. SeqPrep (https://github) was used to remove the adaptor sequences and low-quality sequences (Q <20), sequences containing more than 10% N bases, and sequences less than 20 bases long to obtain clean reads. *C. tinctoria* has no reference genome, and the clean reads were spliced by Trinity (http://trinityrnaseq.sourceforge.net/) to obtain the transcript sequence. The transcript sequence was used as a reference sequence for subsequent analyses. Hierarchical clustering of transcripts was performed by comparing the read number and expression pattern of transcripts using Corset (https://code.google.com/p/corset-project/). Hierarchical clustering was conducted with Corset to ultimately obtain the longest cluster sequence as a follow-up analysis on unigenes (Conesa et al., 2016).

### Functional annotation and classification of unigenes

To annotate and infer the functions of the transcripts, we used BLAST software to compare the unigene sequences with 7 public databases, including the NCBI nonredundant protein (NR, http://www.ncbi.nlm.nih.gov), TrEMBL (a computer annotated supplement to SwissProt), Pfam (http://xfam.org/), SwissProt (http://www.expasy.ch/sprot), and Kyoto Encyclopedia of Genes and Genomes (KEGG) databases (http://www.genome.jp/kegg). Gene Ontology (GO, http://www.geneontology.org) classification based on molecular function (MF), biological process (BP), and cell component (CC) and euKaryotic Ortholog Groups (KOG) classification using the KOG database (http://www.ncbi.nlm.nih.gov/COG/) were also performed.

### Quantitative gene expression and differentially expressed gene (DEG) analysis

The clean reads of 21 samples were mapped back to a reference sequence using Bowtie 2 (http://bowtie-bio.sourceforge.net), and the fragments per kilobase of transcript per million fragments mapped (FPKM) method was used to normalize the expression of mapped reads. The read count of genes was realized using feature counts (Liao, Smyth & Wei, 2014). DESeq2 was used to analyze the difference in read count data. The multiple hypothesis test correction of the *P* value (probability) was carried out using the Benjamini–Hochberg method, and the false discovery rate (FDR) was obtained. Genes that satisfied the conditions of — log_2_FC — ≥ 1 and FDR < 0.05 were differentially expressed. GO enrichment analyses of DEGs were performed using the GO database (Wang, Gerstein & Snyder, 2009; Love, Huber & Anders, 2014).

### Weighted gene coexpression network analysis (WGCNA)

WGCNA is used to find highly related and hub gene modules that play important roles. The present study analyzed the physiological indexes and all DEGs of *C. tinctoria* seedlings under 21 low-salt and high-salt treatments using WGCNA. The first 100 groups of genes with the highest edge weight of WGCNA in the gene module with high correlation with physiological traits were selected and visually analyzed by Cytoscape 3.7.1 to determine the hub gene (Lou et al., 2017).

### QRT-PCR analysis

Total RNA was extracted from leaves using TRIzol reagent (Invitrogen). A NanoDrop 2000 was used to test the concentration and purity of the RNA. A Servicebio RT First-Strand cDNA Synthesis Kit for reverse transcription was used to synthesize cDNA to serve as the template for qRT-PCR. Then, 2 × SYBR Green qPCR Master Mix (High ROX) was used for quantitative PCR (Wuhan Servicebio Technology Co., Ltd). Primers targeting the reference gene *GAPDH* were designed according to the conserved region in a *GAPDH* gene sequence alignment of *Helianthus annuus*. The 2^−ΔΔ*CT*^ method was used to calculate the relative expression of genes. Each gene was examined in three biological replicates. Primer information is shown in the attached Table S1.

### Statistical analysis

An analysis of variance was carried out using SPSS 23.0, and the mean values were separated using the least significant difference (LSD) test at *P* = 0.05. All figures were drawn using Origin 2018. Pearson’s correlation and DEG heat map analyses were achieved via the functions “cor.test” and “heatmap”, respectively.

## Results

### Changes in the physiology and growth of *C. tinctoria* under salt stress

#### Salt stress changed the contents of MDA, proline, soluble sugar and soluble protein in *C. tinctoria* seedlings

**Figure 1:**
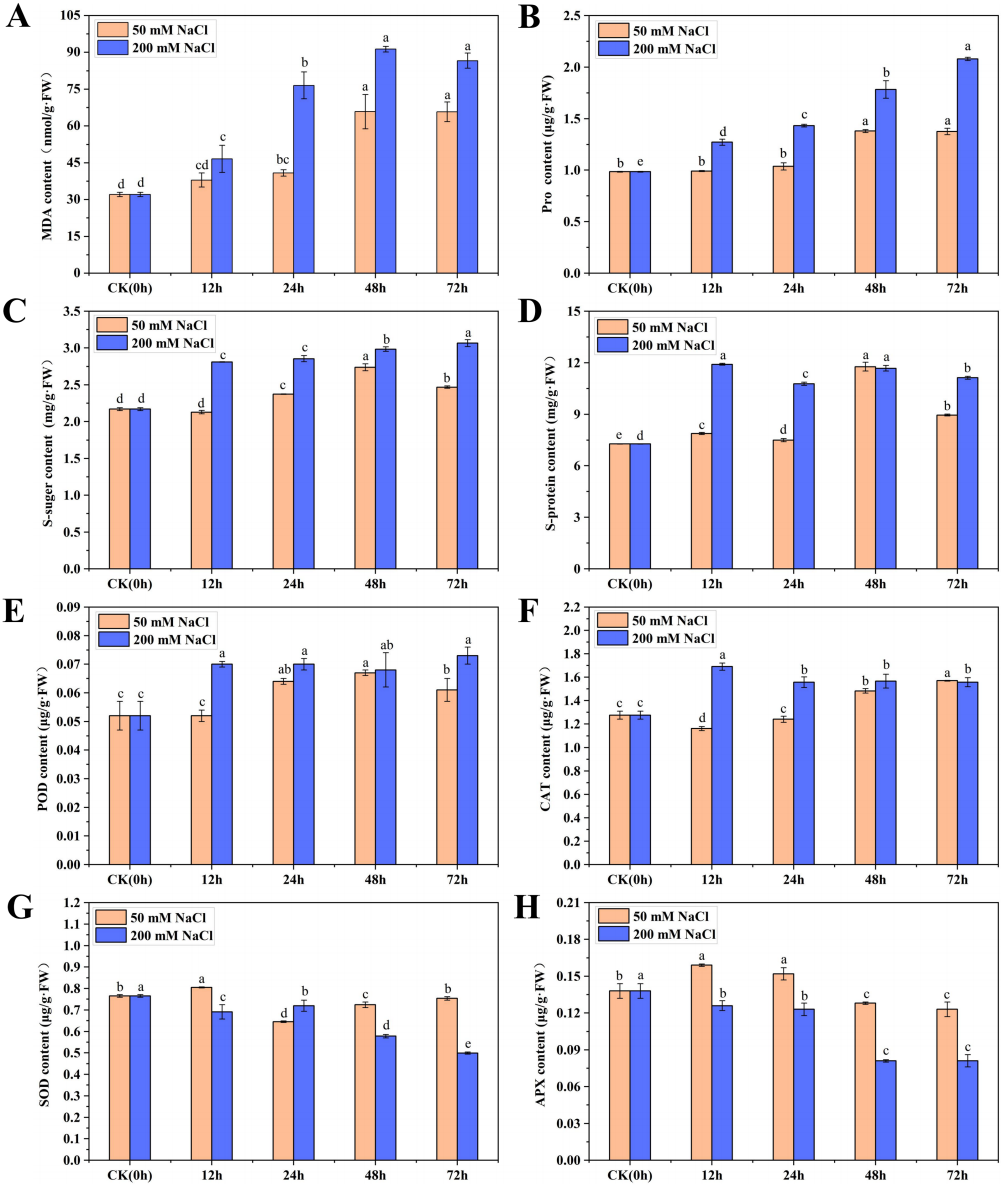
The physiological changes in *C. tinctoria* under 50 and 200 mM NaCl treatments. (A) MDA content; (B) Pro content; (C) Soluble sugar content; (D) Soluble protein content; (E) POD content; (F) CAT content; (G) SOD content; (H) APX content; (A–H) the mean ± SD of three parallel. Different letters correspond to significant differences determined by one-way analysis of variance (ANOVA) (*P* < 0.05).

To understand the physiological and growth changes of *C. tinctoria* under the 50 and 200 mM NaCl treatments, the osmotic regulation substances, antioxidant enzymes and biomass of seedling leaves were quantified within 72 h. The MDA content in the 50 and 200 mM NaCl samples first increased then stabilized over time. The maximum values appeared at 48 h and were 1.05 and 1.87 times higher than that of the CK, respectively (Fig. 1A). The overall change trend of Pro and soluble sugar contents from 0 to 72 h after 50 mM NaCl treatment was the same, with an initial increase followed by a decreasing trend. Their maximum values were reached at 48 h and were 1.40 and 1.26 times higher than CK, respectively. However, the Pro and soluble sugar contents increased gradually after 200 mM NaCl treatments and reached maximum levels at 72 h, which were 1.11 and 0.41 times higher than CK, respectively. The soluble protein content increased at different times after 50 and 200 mM NaCl treatments. Soluble protein increased significantly at 48 h after 50 mM NaCl treatments and was 1.62 times higher than that of the CK. No obvious change in the content of soluble protein was found after 200 mM NaCl treatments, and the content was highest at 12 h and 48 h, which was not significantly different from 50 mM NaCl treatments for 48 h (Figs. 1B–1D). The results showed that the Pro, soluble sugar and soluble protein contents in early *C. tinctoria* seedlings increased significantly under low and high salt stress but increased more under high salt stress, which indicates the positive roles of osmotic and organic regulators in salt stress, especially under high salt stress (200 mM NaCl).

### Salt stress changed the antioxidant enzyme content of *C. tinctoria* seedlings

The change trends of POD in the 50 and 200 mM NaCl treatments increased initially then stabilized with the increase for the 200 mM NaCl samples and was slightly higher than the 50 mM NaCl samples. The change trends of CAT were exactly the opposite. CAT in the 50 mM NaCl treatments decreased at 12 h then gradually increased. However, CAT in the 200 mM NaCl treatment increased at 12 h to a level 1.32 times higher than that of the CK and then decreased to a stable level. Under 50 and 200 mM NaCl treatments, the SOD content increased significantly at 12 h, but the content of other treatments was lower than that of the CK. The APX content increased significantly from 12 to 24 h under 50 mM NaCl treatments and was significantly lower than that of the CK after 48 h. Under 200 mM NaCl treatments, the content of APX was significantly lower than that of the CK at different times (Figs. 1E–1H). Taken together, these results showed that low and high salt stress promoted the activities of POD and CAT to different degrees, and high salt stress significantly inhibited the activities of SOD and APX.

### Salt stress changed the biomass of *C. tinctoria* seedlings

The aboveground fresh weight, underground fresh weight and root/shoot ratio of *C. tinctoria* seedlings were measured 72 h after salt stress. The biomass and root/shoot ratio of *C. tinctoria* seedlings increased to varying degrees under 50 mM NaCl treatments compared to the CK treatment. The aboveground fresh weight, underground fresh weight and root/shoot ratio increased 31.44%, 47.22% and 10.13%, respectively. Under the 200 mM NaCl treatments, the biomass decreased 48.26%, 46.87% and 0.27%, respectively. These results showed that low and high salt significantly affected the growth of *C. tinctoria*. The effect of 50 mM NaCl treatments on the aboveground plant part was less than the underground plant part, and the effect of 200 mM NaCl treatments on the aboveground and underground parts was the same.Low salt (50 mM NaCl) promoted the growth of *C. tinctoria* seedlings, and high salt (200 mM NaCl) significantly inhibited the growth of *C. tinctoria* seedlings (Fig. S1).

### Transcriptome sequencing analysis

To further understand the response of *C. tinctoria* morifolium seedlings to low and high salt treatments, *de novo* transcriptome sequencing was performed on the leaves of *C. tinctoria* after 50 (FS) and 200 (TS) mM NaCl treatments for 0 (CK), 12 h, 24 h and 48 h. cDNA libraries of 21 transcriptome sequences were constructed. For each sample, 41,630,370–50,805,280 raw reads were obtained from high-throughput sequencing, and after splices and low-quality sequences were excluded, 40,125,080–49,057,376 clean reads were obtained. Q30 bases accounted for greater than 93% of the total, and the average GC content was 45.49%. The data for each sample are summarized in Table S2. After assembly, 507,230 transcripts with an average length of 678 bp and 428,638 unigenes with an average length of 750 bp were identified. The N50 values were 864 bp and 922 bp, respectively (Table S3). A total of 14,588 transcripts and unigenes, with an average sequence length of more than 2,000 bp, were found (Fig. S2).

### Comments and functional classification of unigenes

We compared a total of 428,638 unigenes with information from 7 public databases (the NR, TrEMBL, SwissProt, Pfam, KOG, GO, and KEGG databases) and found that all unigenes shared a similarity of 63.76% in at least one database (Table S4). A comparison of 270,860 genes with the NR database showed that 76.31% (206,689) of the genes had the greatest sequence similarity with *Helianthus annuus* and low homology with other species (Fig. S3A). Based on the classification of 227,019 genes successfully annotated to one or more GO terms in the GO database (Table S3), all genes were divided into 3 GO categories and 59 groups. The most obvious matching GO terms in the “biological process” category were cellular process (134,866), metabolic process (115,375) and response to stimulus (60,937). The main terms in “cell components” were cell (154,999), cell part (154,612), and organelle (117,183). For the category of “molecular function”, GO binding (136,084), catalytic activity (121,727), and transporter activity (14,544) had the largest number of genes (Fig. S3B). The most common homologous groups were general function prediction only (33,064), signal transduction mechanisms (17429), posttranslational modification, protein turnover, chaperones (15,911) and transcription (8,049) in the KOG database for linear gene classification and functional prediction of 146589 unigenes (34.20%) (Fig. S3C). We further mapped unigenes (203,045) to the KEGG database for annotation and enrichment analysis. All genes were divided into five biochemical pathways, cellular processes, environmental information processing, genetic information processing, metabolism, and organismal systems, with the largest proportion of unigenes in the metabolic pathway. The metabolic pathway terms metabolic pathways (38,578), biosynthesis of secondary metabolites (18,406) and carbon metabolism (5,186) involved the largest number of genes (Fig. S3D).

### Gene expression and DEG analysis

To evaluate the overall trends of gene expression, we used a box plot with the expression level in terms of FPKM on the horizontal axis. The expression levels of most genes were consistent, and only a few genes were distinct (Fig. S4A). The Pearson’s correlation coefficients of the expression levels of each library showed a high correlation among biological repeats in each group of 50 and 200 mM NaCl treatment treatments (R^2^ > 0.9) and a significant difference in gene expression between groups (Fig. S4B).

**Figure 2:**
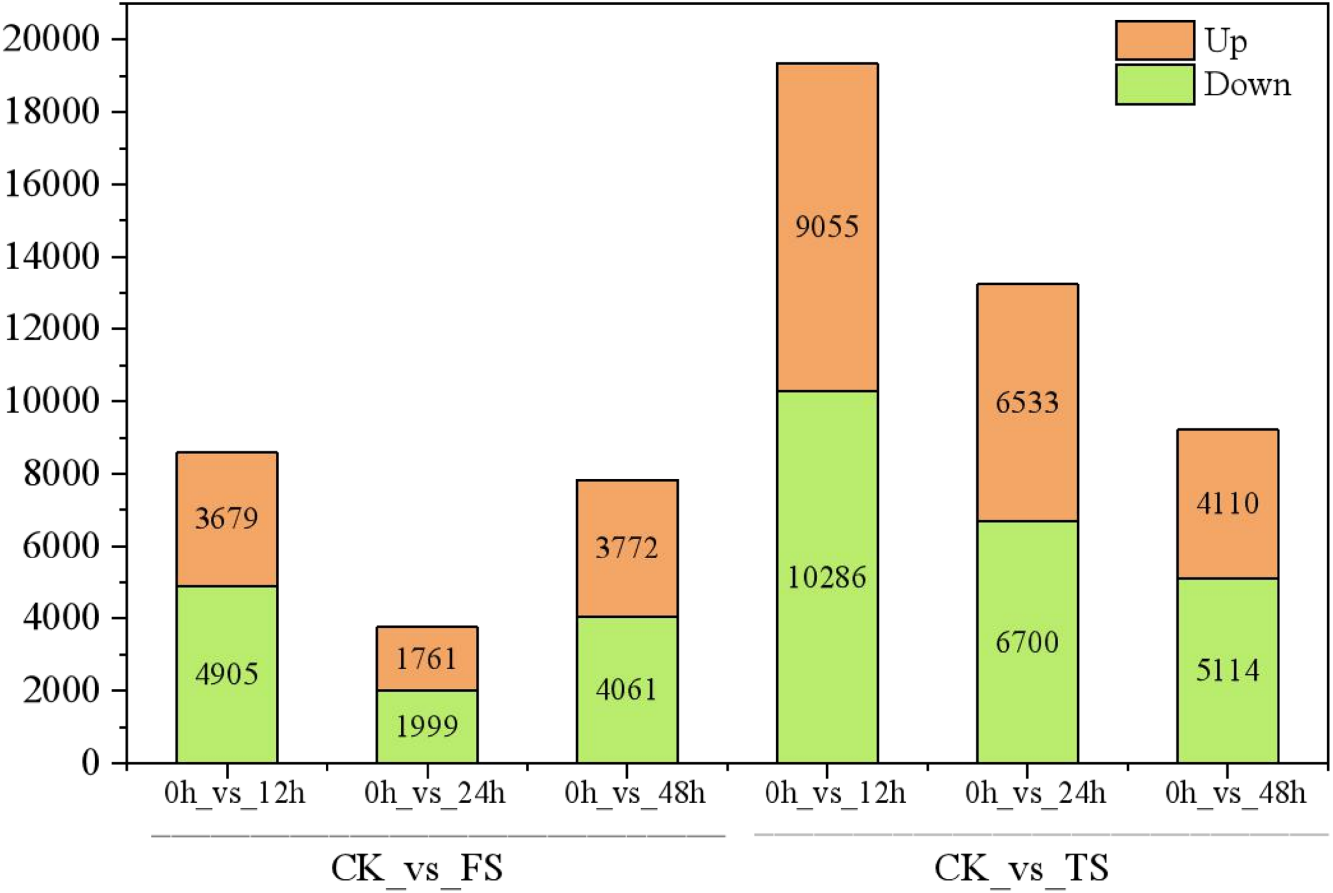
Statistics of DEGs in leaves of *C. tinctoria*. FS:50 mM NaCl treatments;TS:200 mM NaCl treatments;Green indicates the number of downregulated genes, and orange indicates the number of upregulated genes.

**Figure 3:**
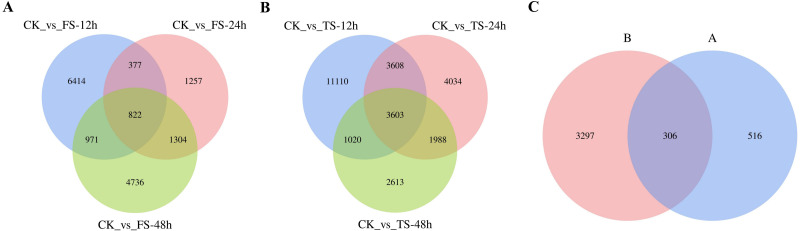
Venn diagrams of DEGs. (A) the coexpressed and specifically expressed DEGs under the compared groups of CK vs 50 mM NaCl treatments; (B) the coexpressed and specifically expressed DEGs under the compared groups of CK vs 200 mM NaCl treatments; (C) the coexpressed and specifically expressed DEGs between A vs B; FS:50 mM NaCl treatments; TS: 200 mM NaCl treatments.

The selected DEGs (— log_2_FC) — ≥ 1 and FDR < 0.05) were then compared and analyzed. In total, 3,679, 1,761, 3,772, 9,055, 6,533, and 4,110 upregulated genes and 4,905, 1,999, 4,061, 10,286, 6,700 and 5,114 downregulated genes at 12 h, 24 h and 48 h for the 50 and 200 mM NaCl treatments compared with the CK treatmen were detected (Fig. 2). The number of DEGs under 200 mM NaCl treatments decreased gradually over time, and the number of DEGs under 50 mM NaCl treatments decreased initially then increased (Fig. 2). The number of DEGs peaked at 12 h in both treatments. DEG analysis showed that the DEGs were downregulated slightly under 50 and 200 mM NaCl treatments, and the gene expression changed more actively within 24 h under the 200 mM NaCl treatment (Fig. 2). A Venn diagram showed that 3,603 DEGs were expressed under 200 mM NaCl treatments at different times (Fig. 3A), which was much higher than the coexpressed DEGs in the 50 mM NaCl treatment groups (Fig. 3B). A total of 306 DEGs were expressed in all the comparison groups (Fig. 3C).

### Identification and functional annotation of the physiological indicators related to DEGs by WGCNA

**Figure 4:**
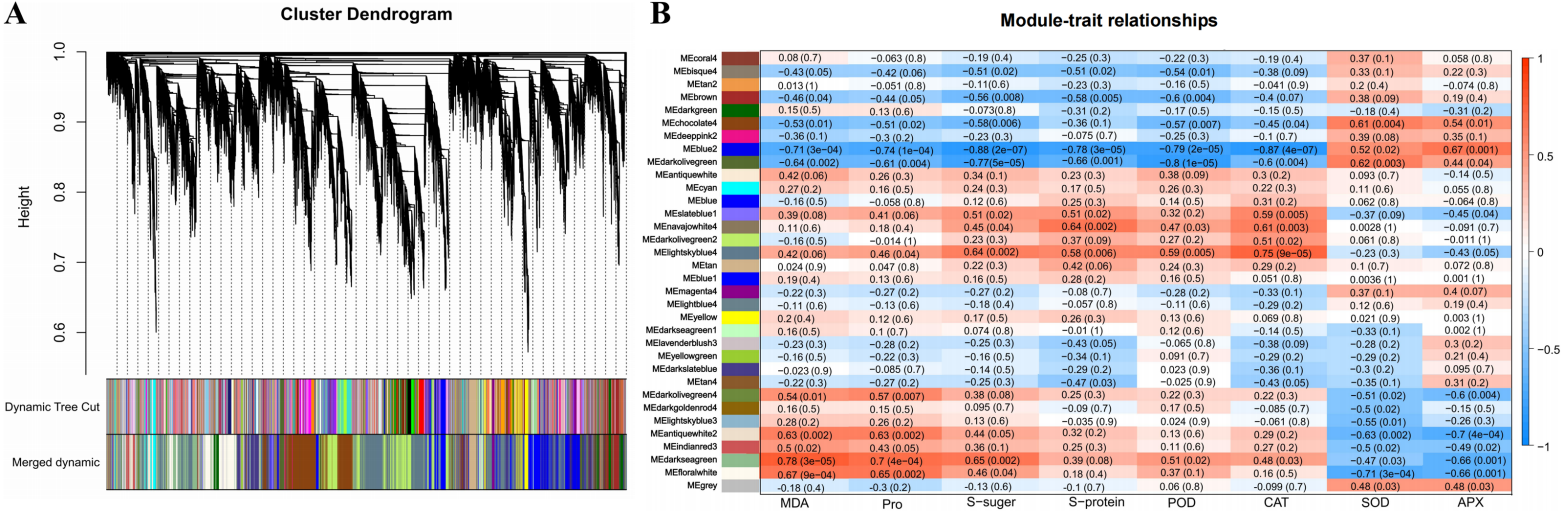
Weighted gene coexpression network analysis (WGCNA) of all DEGs and physiological index (pearson correlation coefficient, PCC > 0.8 or < −0.8). (A) Hierarchical clustering tree (cluster dendrogram) showing 34 modules of co-expressed genes by WGCNA. Each leaf of tree corresponds to one gene. The major tree branches constitute 34 modules, labeled with different colors. (B) Module-physiological indexes relationship. Each row represents a module, and the number of genes in each module is shown on the left. Each column represents a specific physiological index. The value in each cell at the row-column intersection represents the correlation coefficient between the module and the physiological index and is displayed according to the color scale on the right. The value in each cell represents the *P*-value.

A total of 34 DEG modules were identified by WGCNA, four of which (blue2, Darkolivegreen, Lightskyblue4 and Darkseagreen) highly correlated with physiological indicators (Pearson’s r > 0.7 or r < −0.7, *p* value < 0.01) (Figs. 4A–4B). To determine the transcriptional expression of these modules, heat maps of the genes in these four modules were drawn (Fig. S5). Under salt stress, the genes in module blue2 had important effects on Pro (*r* = −0.74, *p* value = 1.0 ×10^−4^), soluble sugar (*r* = - 0.88, *p* value = 2.0 ×10^−7^), soluble protein (*r* = −0.78, *p* value = 3.0 ×10^−5^) and POD (*r* = −0.79, *p* value = 2.0 ×10 ^−5^). The heat map of the gene expression in the blue2 module showed that gene expression in the CK and 50 mM NaCl treatments was significantly higher than that in the 200 mM NaCl treatment (Fig. 4B, Fig. S5A), which indicated that blue2 was a specific coexpression module in response to low salt stress. The Darkolivegreen module highly negatively correlated with soluble sugar (*r* = −0.77, *p* value = 5.0 ×10^−5^) and POD (r = −0.80, *p* value = 1.0 ×10^−5^). The expression of genes in this module was the highest in the CK and 50 mM NaCl treatment groups (Fig. 4B, Fig. S5B). The Darkseagreen module highly positively correlated with MDA (*r* = 0.78, *p* value = 3.0 ×10 ^−5^). The heat map of gene expression indicated that the gene expression levels in 200 mM NaCl groups after 24 h and 48 h of treatment were higher than the other treatments (Fig. 4B, Fig. S5C). The genes in the Lightskyblue4 module had an important influence on CAT (*r* = 0.75, *p* value = 9.0 ×10^−5^). The expression of genes in Lightskyblue4 under 200 mM NaCl treatments at different times was significantly higher than that under 50 mM NaCl treatments (Fig. 4B, Fig. S5D), which indicates that Lightskyblue4 was a specific coexpression module in response to high salt stress.

**Figure 5:**
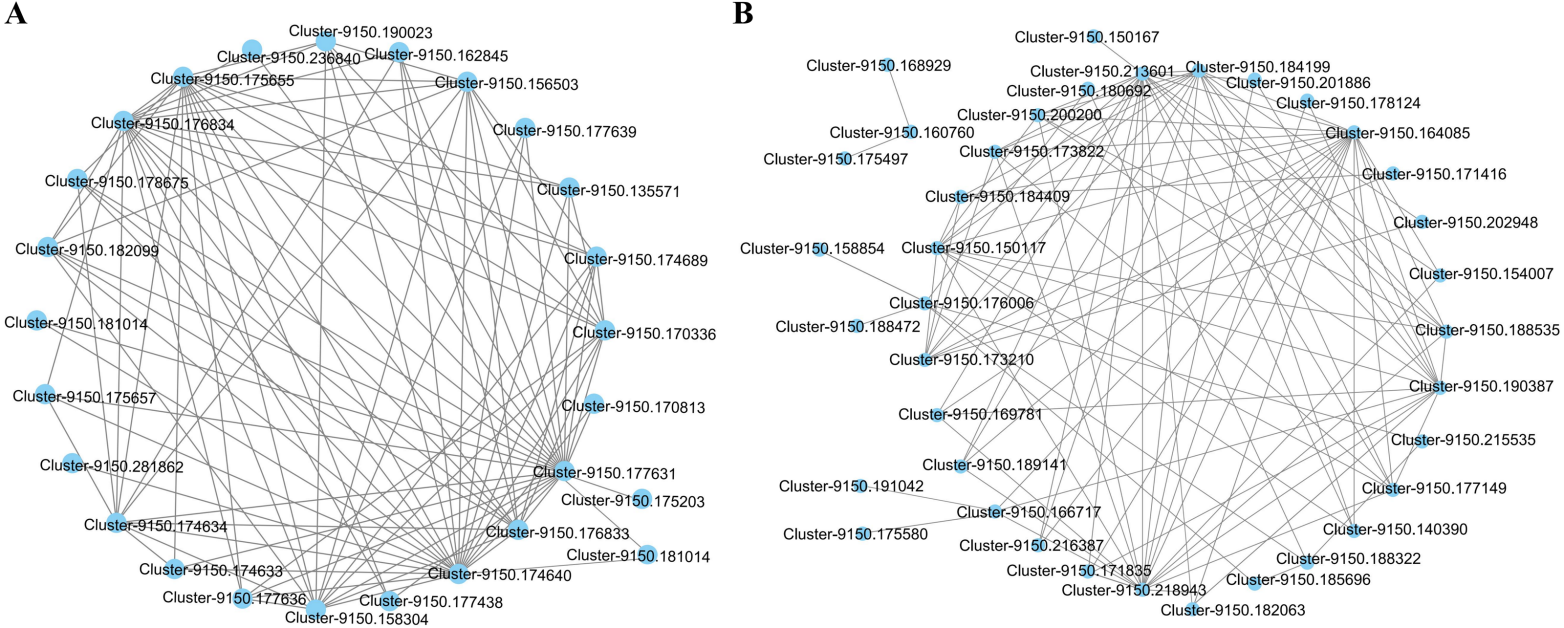
The network of the top 100 co-expressed genes pairs from WGCNA in two modules. The WGCNA (weighted gene co-expression network analysis) used all DEGs with *P* < 0.05 in whole samples. (A) 25 DEGs blue2 module. (B) 37 genes highly connected in the Lightskyblue4 module.

The interaction network of the top 100 genes with the highest weight of WGCNA that were specific to the blue2 and Lightskyblue4 modules was visualized using Cytoscape. Twenty-five genes with WGCNA weights > 0.19 were highly connected in the blue2 module, and 9 genes had more than 10 edges (Fig. 5A). Thirty-seven genes with WGCNA weights > 0.31 were highly connected in the Lightskyblue4 module, and 6 genes had more than 10 edges (Fig. 5B).

To explore the biological functions of these genes, the most significantly enriched GO terms in the blue2 and Lightskyblue4 modules were first identified (Table S5). Five BP GO terms, including reductive pentose-phosphate cycle, photosynthesis, dark reaction, photosynthetic electron transport chain, photosynthetic electron transport in photosystem I and plastid membrane organization, were significantly enriched in the blue2 module. The annotation of the 25 genes is shown in Table S6, and it mainly includes 15 enzyme genes, 5 protein-related genes, and 5 genes with unknown functions. The genes in the Lightskyblue4 module were significantly enriched in proline biosynthetic process, L-proline biosynthetic process, proline metabolic process, peptidyl-threonine phosphorylation, and peptidyl-threonine modification. The annotations of 37 genes, including 22 enzyme genes, 8 protein-related genes and 7 genes with unknown functions, are shown in Table S7.

### Related genes responding to physiological changes in *C. tinctoria* under salt stress

**Figure 6:**
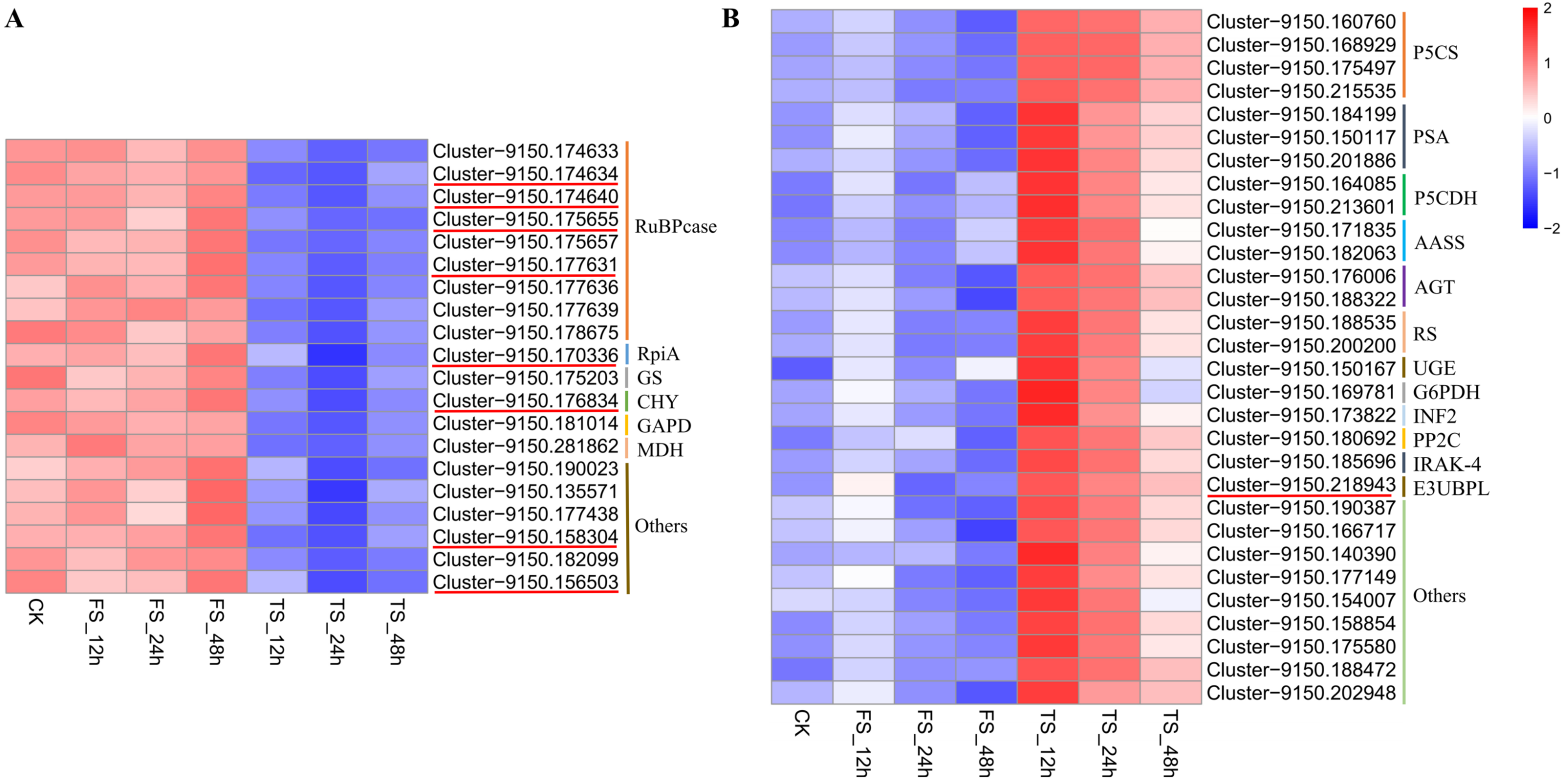
Heatmap of hub gene. (A) the heatmap of 20 hub genes in the blue2 module; (B) the heatmap of 30 hub genes in the Lightblue4 module. FS: 50 mM NaCl treatments; TS: 200 mM NaCl treatments.

Twenty and 30 coexpressed hub genes related to physiological changes in the blue2 and Lightsskyblue4 modules were screened, respectively. In the blue2 module, 9 *RuBPcase*, 1 *RpiA*, 1 *GS*, 1 *CHY*, 1 *GAPD*, and 1 *MDH* genes were found to be involved in the regulation of *C. tinctoria* metabolism and other biological processes. In the Lightskyblue4 module, 4 *P5CS* encoding key enzymes, 3 *PSA*, 2 *P5CDH*, 2 *RS*, 2 *AASS*, 2 *AGT*, 1 *UGE*, 1 *G6PDH*, 1 *INF2*, 1 *PP2C*, 1 *IRAK-4*, and 1 *E3-UBPL* genes were detected. These DEGs played important regulatory roles in the antioxidant system under high salt (200 mM NaCl) conditions. The hub gene expression of 20 genes in blue2 and 30 genes in Lightskyblue4 that were related to changes in physiological indicators are shown in Fig. 6. The genes in the blue2 module were highly expressed in CK and under the 50 mM NaCl treatment and included 4 *RuBPcase*, 1 *RpiA* and 1 *CHY* genes located in the core position of the network diagram. These genes were expressed at a higher level after 48 h of treatment. The accumulation of physiological indicators (except SOD) that were highly related to the blue2 module in the 200 mM NaCl treatment group was higher than that in the 50 mM NaCl treatment group. However, the total expression of the hub genes was contrary to the gene accumulation, which indicates a negative regulatory relationship between them. Genes in Lightskyblue4 were highly expressed under 200 mM NaCl treatments, especially after 12 h of treatment. Among these genes, 2 *PSA*, 2 *P5CDH* and 1 *E3UBP* genes were in the core position of the network diagram. The accumulation of the total expression of these key genes in the 200 mM NaCl treatments was significantly higher than that in the 50 mM NaCl treatments, which was consistent with the content changes in CAT (Fig. 6).

### Verification of gene expression by qRT-PCR

To confirm the expression patterns of DEGs obtained from RNA-seq, 12 DEGs involved in flavonoid biosynthesis were randomly selected for analysis by real-time quantitative reverse transcription PCR (qRT-PCR) (Fig. S6). For all selected genes, the expression trend from qRT-PCR was consistent with that from the RNA-seq data, which indicates that the RNA-seq data re reliable.

## Discussion

Salt damage to plants mainly includes osmotic injury and specific ion toxicity that affect a wide variety of physiological and metabolic processes in plants (Gu et al., 2016). To cope with saline environments, plants adapt to salt stress by regulating morphology, physiology and gene expression (Li, Xu & Wang, 2020). To explore the salt adaptability of *C. tinctoria*, we analyzed the response of *C. tinctoria* to low and high salt stress by combining physiological and transcriptome data.

Under salinity conditions, the production of organic osmotic protective agents is essential for higher plants to adapt to the salt environment (Yan et al., 2013). Previous studies on Atriplex canescens (Guo et al., 2019), oat (Gao et al., 2015), *Chenopodium quinoa* Willd (Hajihashemi et al., 2020) showed that the accumulation of a large number of affinity solutes (including Pro, glycine, betaine, soluble sugar and soluble protein) was an important mechanism for plants to adapt to salinization conditions. In this study, Pro, soluble sugar and soluble protein accumulated to varying degrees under 50 and 200 mM NaCl conditions and increased more in the 200 mM NaCl treatments (Figs. 1B–1D). These results are similar to previous studies. The accumulation of Pro under salt stress mainly occurs via the glutamate pathway. NH4^+^ iscatalyzed into glutamine by (glutamine synthetase) *GS*, which is catalyzed by glutamate synthase (GOGAT) to generate glutamate (Glu). Glu is directly produced under the action of delta-1-pyrroline-5-carboxylate synthetase (*P5CS*) and other enzymes in plants. Therefore, the formation of Pro is related to *GS* and *P5CS* (Zhao et al., 2014). Li et al. (1999) believed that a certain concentration of NaCl treatments inhibited the expression of *GS. GS* (Cluster-9150.175203) was highly expressed in the CK and 50 mM NaCl treatments in the present study and decreased significantly under the 200 mM NaCl treatment, which was similar to the results of Li. The expression of 4 *P5CS* genes (Cluster-9150.160760, Cluster-9150.168929, Cluster-9150.175497, and Cluster-9150.215535) was significantly high under 200 mM NaCl treatments, unlike the expression of *GS*. These results are similar to Hu’s finding that *P5CS* expression in pea roots was significantly increased under salt stress (Hu, Delaueny & Verma, 1992). Under high salt stress, the increase in Pro accelerated, which might be the result of high salt stress stimulating *C. tinctoria* to** quickly start the *P5CS* gene encoding Pro synthesis (Huang et al., 2013; Özge Çelik & Atak, 2012). Previous studies have also found that the degradation of proline played an important role in the scavenging process. In the study of Arabidopsis thaliana, it was found that the P5C-Pro cycle between cytoplasm and mitochondria was related to 1-pyrroline-5-carboxylate dehydrogenase (*P5CDH*) activity, and the oxidation process from delta-1-pyrroline-5-carboxylate (P5C) to Glu catalyzed by *P5CDH* plays a key role in avoiding the excessive production of reactive oxygen species (ROS) (Deuschle et al., 2004). In this study, 2 *P5CDH* genes (Cluster-9150.164085 and Cluster-9150.213601) were highly expressed under 200 mM NaCl treatments, it showed that the catalysis of the 2 *P5CDH* overexpression had a direct effect on scavenging ROS in the process of proline degradation. The galactoside synthase (*GS*) and raffinose synthase (*RS*) are the main enzymes regulating the metabolism of these oligosaccharides. *GS* catalyzes the synthesis of galactoside inositol, and its catalytic reaction is the rate limit of oligosaccharide accumulation. The gene expression of *GS* is related to the accumulation of raffinose oligosaccharides. *RS* is involved in regulating the conversion of sucrose to raffinose and inducing the synthesis of stachyose and verbascose. Raffinose and its homolog stachyose are soluble sugars in many plants and participate in sugar transportation in the phloem (Keunen et al., 2013; Li & Xiao, 2008). The total expression of *GS* (Cluster-9150.175203) under 50 mM NaCl treatments was significantly higher than the 200 mM NaCl treatments in the present study. However, the expression level of 2 *RS* genes (Cluster-9150.188535, Cluster-9150.200200) was opposite of *GS.* These results suggest that inositol galactoside and raffinose are osmotic protective agents under salt stress, and *GS* and *RS* play an important role in inositol galactoside and raffinose accumulation under low and high salt stress, respectively. We also found that glucose-6-phosphate 1-dehydrogenase (*G6PDH,* Cluster 9150.169781) and ribose 5-phosphate isomerase A (*RpiA*, Cluster 9150.170336), which are involved in the pentose phosphate pathway (PPP), were highly expressed in the 200 and 50 mM NaCl treatments, respectively. Yasue & Tetsuo (2000) found that salt stress induced the expression of *G6PDH* in wheat, and the expression decreased over time, which is consistent with the results of the present study. *RpiA* is involved in the reversible isomerization reaction of ribose-5-phosphate (R5P) and ribulose-5-phosphate (Ru5P). This reversible reaction ensures the flexibility of the cell demand for glucose metabolic intermediates and may be related to adaptability to saline environments.

Salt stress causes excessive production of ROS in plant cells, including singlet oxygen, superoxide anion (O^2−^), hydrogen peroxide (H_2_O_2_), and hydroxide ion (HO^−^). Antioxidant enzyme systems (*e.g*., SOD, CAT, APX and GR) are important defense systems for plants under salt environmental stress (Yang et al., 2016). The main function of CAT is the removal of the H_2_O_2_ that is produced in the process of β-fatty acid oxidation and photorespiration. Plants regulate changes in H_2_O_2_ by adjusting the CAT content in cells under stress, which prevents the damage caused by reactive oxygen free radicals (Liu et al., 2019). CAT increased to varying degrees under 50 and 200 mM NaCl treatments in the present study, but it decreased from 24 h in 200 mM NaCl treatments (Fig. 1F). This result indicates a mechanism for increasing defense against ROS via CAT in *C. tinctoria* within a certain range of salinity levels. CAT in plants mainly exists in peroxisomes and glyoxylic acid cycle bodies, and it is closely related to chloroplasts and mitochondria. Nine ribulose bisphosphate carboxylase (*RuBPcase*) genes were coexpressed in chloroplasts in each treatment. RuBPcase accounts for greater than 50% of the total soluble protein in chloroplasts, and it catalyzes carboxylation and oxygenation reactions. The carboxylation of 1,5-diphosphate ribulose (RuBP) to form 3-phosphoglycerate and the oxygenation of RuBP to form phosphoglycolic acid and 3-phosphoglyceric acid are related to energy metabolism. The glycolic acid formed by the oxygen reaction is the substrate for photorespiration. RuBPcase catalyzes the rate of the oxygenation reaction, which is directly related to photorespiration. More H_2_O_2_ might be produced in the photorespiration process and induce a change in CAT content. The total expression of the 4 *RuBPcases* (Cluster-9150.174634, Cluster-9150.174640, Cluster-9150.175655, and Cluster-9150.177631) with higher connectivity in 50 mM NaCl treatments was higher than under high salt stress in the present study, which suggests that salt stress affects the rate of the catalytic oxygenation reaction of RuBPcases and indirectly affects the change in CAT content. A large number of studies showed that salt stress induced an increase in POD activity (Yang et al., 2016), and similar results were obtained in the present study. The POD content accumulated in the 50 and 200 mM NaCl treatment groups, especially in the high salt treatments. In addition, 2 puromycin-sensitive aminopeptidase (*PSA*) genes (Cluster-9150.184199 and Cluster-9150.150117) involved in the hydrolysis of a variety of proteins were also discovered. *PSA* is essential for cell growth and viability (Eugenio Saénchez-Moraén et al., 2004), but whether it plays a role in salt stress requires further study. The UDP-glucose 4-epimerase (*UGE*) and 3-hydroxyisobutyryl-CoA hydrolase (*CHY*) genes in Arabidopsis have been shown to demonstrate stress responses under adverse conditions and cold stress, respectively. One *UGE* gene (Cluster-9150.150167) was highly expressed under high salt stress in the present study, and 1 *CHY* gene (Cluster-9150.176834) was highly expressed under low salt stress, which indicates that these genes play an important role in adversity (Gipson et al., 2017; Rösti et al., 2007).

## Conclusions

The physiological changes and gene expression of *C. tinctoria* at different low-salt and high-salt treatment times were analyzed. Analysis of the correlation between physiological indicators and the transcriptome identified some candidate genes related to the accumulation of proline, soluble sugar and CAT, which might be related to salt tolerance. The accumulation of soluble sugar and proline under salt stress, the response to the antioxidant enzyme system, and the changes in related gene expression might be strategies for *C. tinctoria* to adapt to and avoid more serious damage from salt stress. However, research on the salt tolerance of *C. tinctoria* is limited. The salt tolerance of *C. tinctoria* involves complex regulation and signal transduction mechanisms. How these candidate genes participate in the salt stress response of snow chrysanthemum needs further study.

##  Supplemental Information

10.7717/peerj.11888/supp-1 Supplemental Information 1 Changes of biomass under salt stress

10.7717/peerj.11888/supp-2 Supplemental Information 2 *De novo* assembly length distribution of sequences

10.7717/peerj.11888/supp-3 Supplemental Information 3 The annotation of unigenes (A) NR annotated pie chart of unigenes. (B) GO functional classification of assembled unigenes. A total of 227,019 unigenes are assigned to at least one GO term and grouped into 3 main GO categories and 59 groups, 28 groups in “biological process” domain, 18 in “cellular component” domain, and 13 in “molecular function” domain, respectively. The abscissa are Go categories, and the ordinate indicates the number of unigenes. (C) KOG functional classification of assembled unigenes.163,272 unigenes were aligned to 25 KOG groups. The abscissa are KOG categories, and the ordinate indicates the number of unigenes. (D) Functional classification and pathway assignment of assembled unigenes by KEGG. A total of 203,045 unigenes are classified to the 5 main KEGG categories, abscissa indicates the ratio of annotated unigenes, and the ordinate is name of KEGG metabolic pathway.

10.7717/peerj.11888/supp-4 Supplemental Information 4 Gene expression level and correlation of 21 samples (A) FPKM expression levels distribution box plot for each group. The abscissa represents different samples, the ordinate represents the logarithmic value of the sample expression FPKM. (B) Correlation heatmap of 21 samples. The numbers in the figure indicate *R*^2^. The closer *R*^2^ is to 1, the stronger the correlation between the two replicate samples. The depth of the color indicates the strength of the correlation. FS:50 mM NaCl treatments; TS:200 mM NaCl treatments.

10.7717/peerj.11888/supp-5 Supplemental Information 5 Gene expression heat maps of four modules with strong correlation with physiological indexes

10.7717/peerj.11888/supp-6 Supplemental Information 6 Quantitative RT-PCR validations A total of 12 genes were selected for qRT-PCR analysis. The GAPDH gene was chosen as the reference gene. FS:50 mM NaCl treatments; TS:200 mM NaCl treatments.

10.7717/peerj.11888/supp-7 Supplemental Information 7 Primer information

10.7717/peerj.11888/supp-8 Supplemental Information 8 Summary of Illumina transcriptome sequencing analysis

10.7717/peerj.11888/supp-9 Supplemental Information 9 Summary of the sequence assembly results

10.7717/peerj.11888/supp-10 Supplemental Information 10 Summary of functional annotation for assembled unigenes

10.7717/peerj.11888/supp-11 Supplemental Information 11 The first 10 GO terms with significant enrichment of blue2 and Lightskyblue4 modules

10.7717/peerj.11888/supp-12 Supplemental Information 12 Description of 25 genes in the blue2 module

10.7717/peerj.11888/supp-13 Supplemental Information 13 Description of 37 genes in the Lightskyblue4 module

10.7717/peerj.11888/supp-14 Supplemental Information 14 Figs. 1 and 6 data

## Additional Information and Declarations

### DNA Deposition

The following information was supplied regarding the deposition of DNA sequences: The sequences are available at GenBank: https://www.ncbi.nlm.nih.gov/bioproject/PRJNA668289. The transcriptome assembly is available in the TSA database: 1949466950.
https://www.ncbi.nlm.nih.gov/nuccore/1949466950
https://www.ncbi.nlm.nih.gov/bioproject/PRJNA668289.

### Data Availability

The following information was supplied regarding data availability: The sequences are available at GenBank: PRJNA668289. The transcriptome assembly is available in the TSA database: 1949466950.
