# Supplementary figures and images for: Physiological changes and transcript identification in Coreopsis tinctoria Nutt. in early stages of salt stress
Source: PeerJ. 2021 Aug 9;9:e11888. doi: 10.7717/peerj.11888 (PMC8359800; doi:10.7717/peerj.11888)

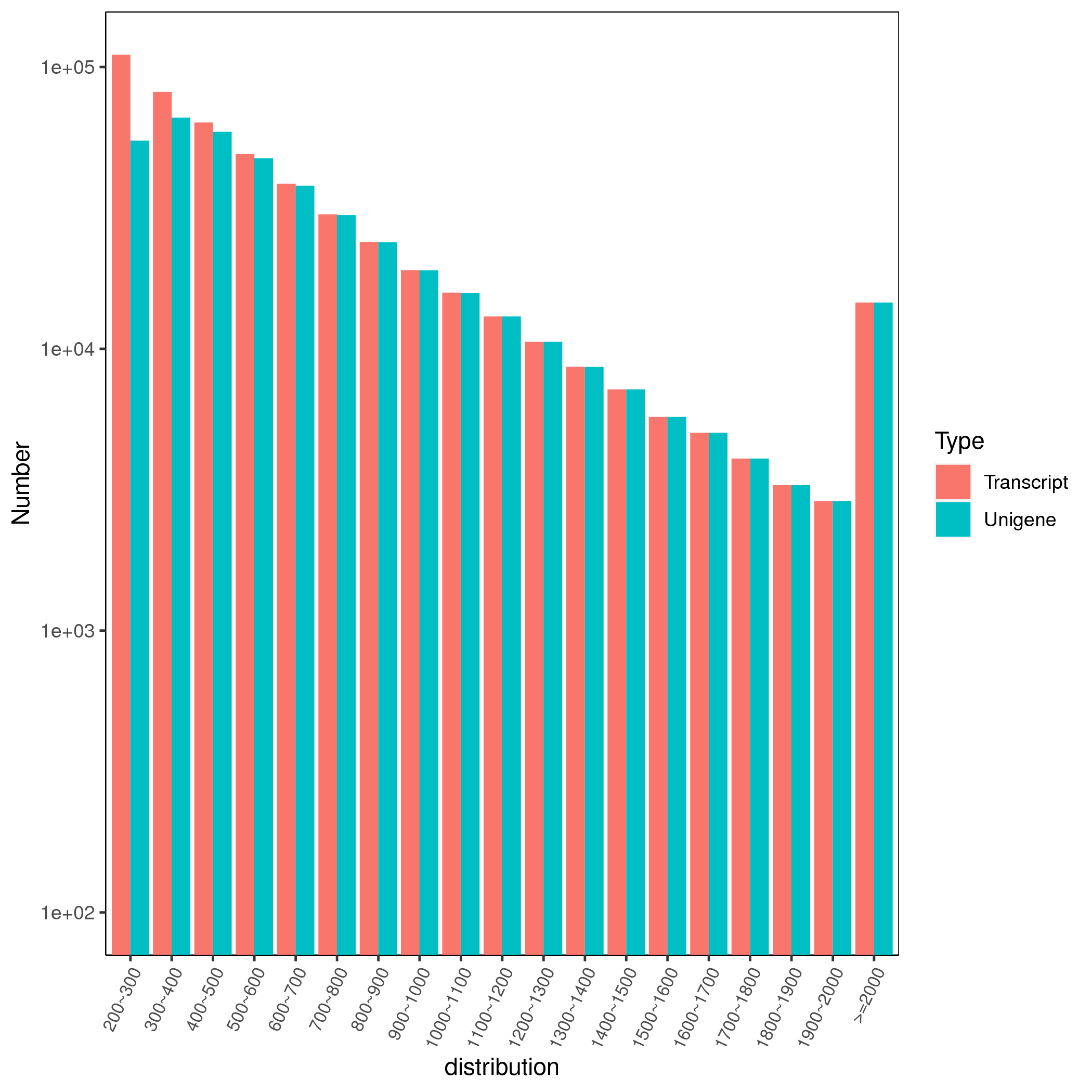

Supplement: Supplemental Information 1 [file peerj-09-11888-s001.png]

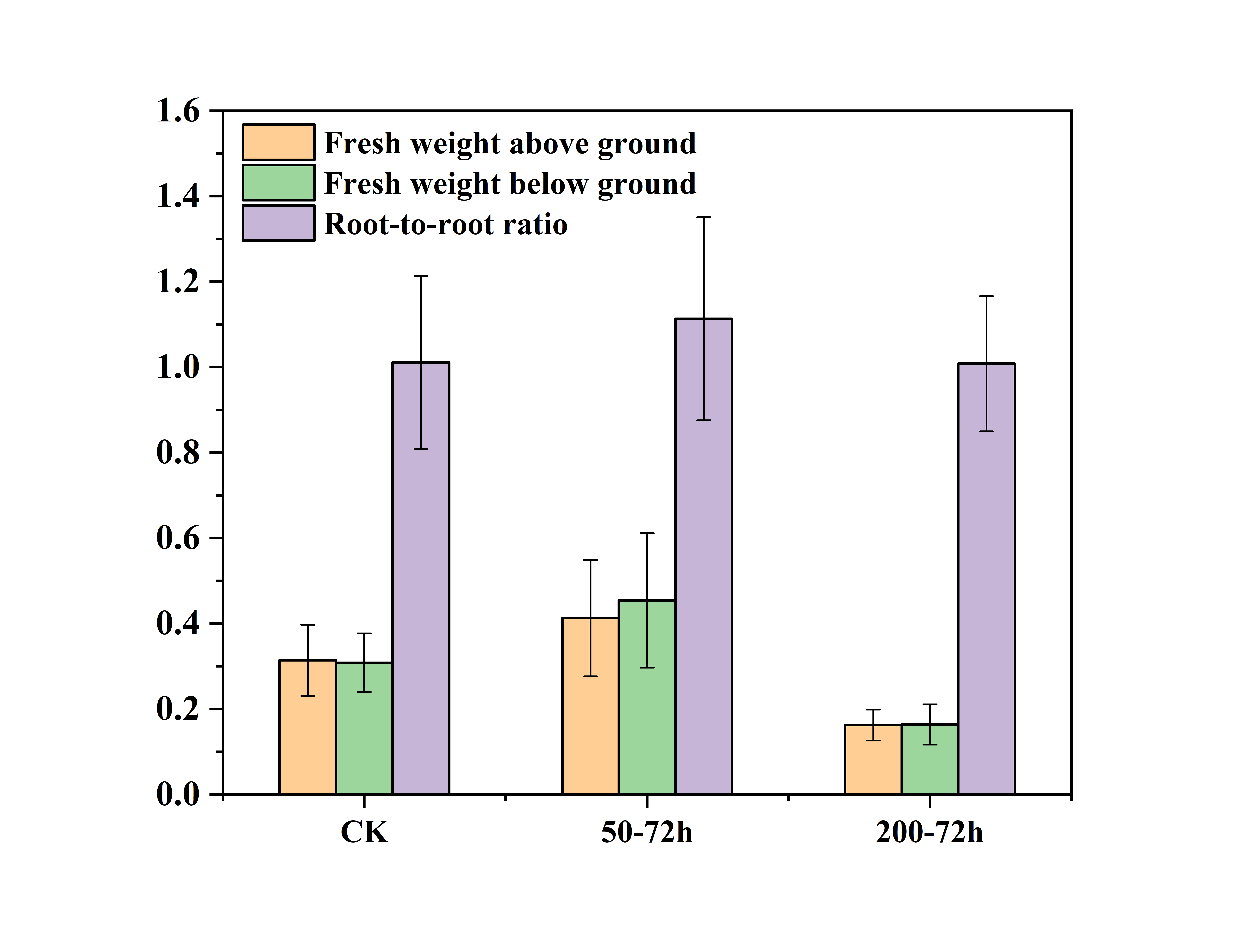

Supplement: Supplemental Information 2 [file peerj-09-11888-s002.png]

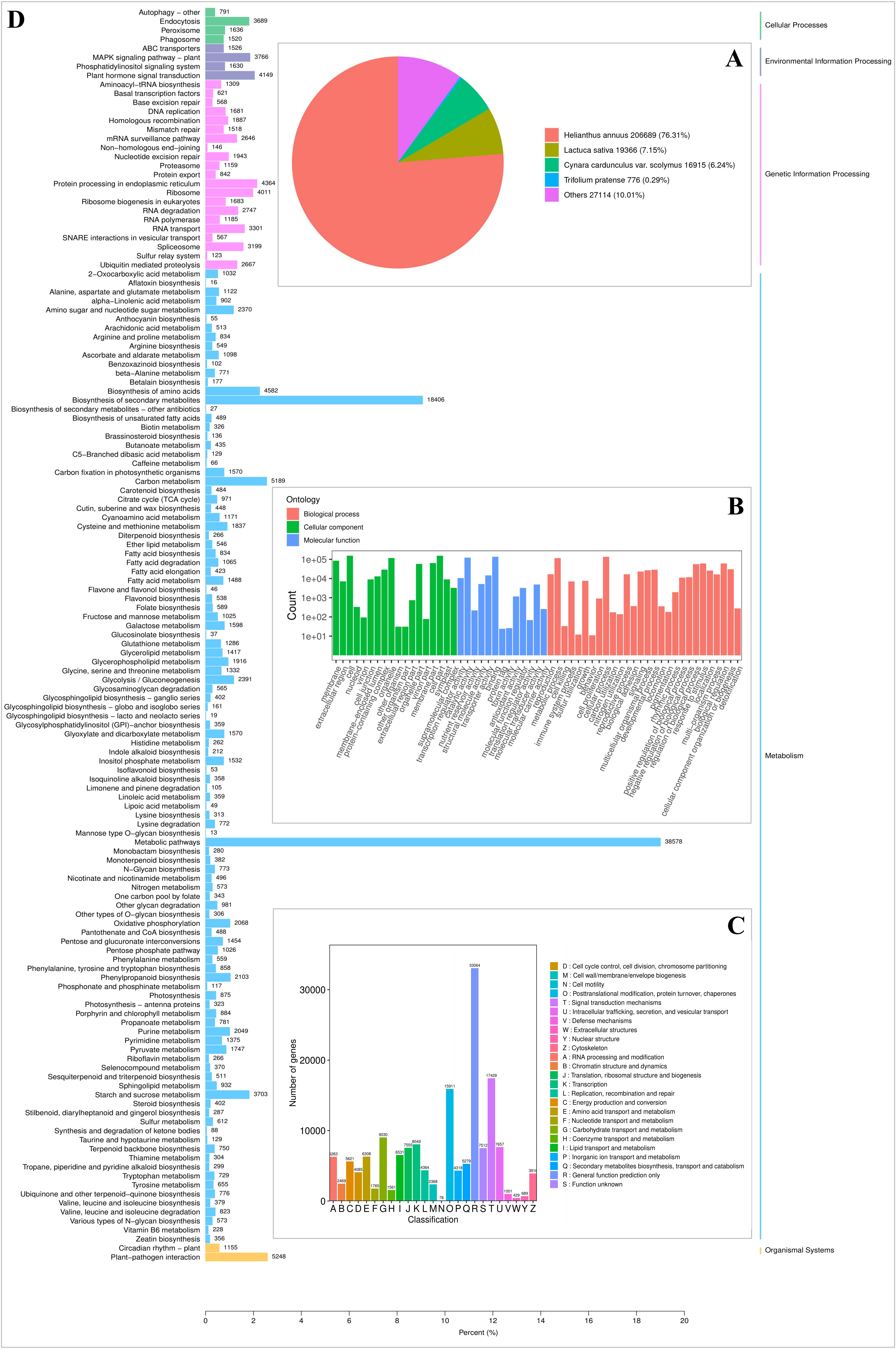

Supplement: Supplemental Information 3 — (A) NR annotated pie chart of unigenes. (B) GO functional classification of assembled unigenes. A total of 227,019 unigenes are assigned to at least one GO term and grouped into 3 main GO categories and 59 groups, 28 groups in “biological process” domain, 18 in “cellular component” domain, and 13 in “molecular function” domain, respectively. The abscissa are Go categories, and the ordinate indicates the number of unigenes. (C) KOG functional classification of assembled unigenes.163,272 unigenes were aligned to 25 KOG groups. The abscissa are KOG categories, and the ordinate indicates the number of unigenes. (D) Functional classification and pathway assignment of assembled unigenes by KEGG. A total of 203,045 unigenes are classified to the 5 main KEGG categories, abscissa indicates the ratio of annotated unigenes, and the ordinate is name of KEGG metabolic pathway. [file peerj-09-11888-s003.png]

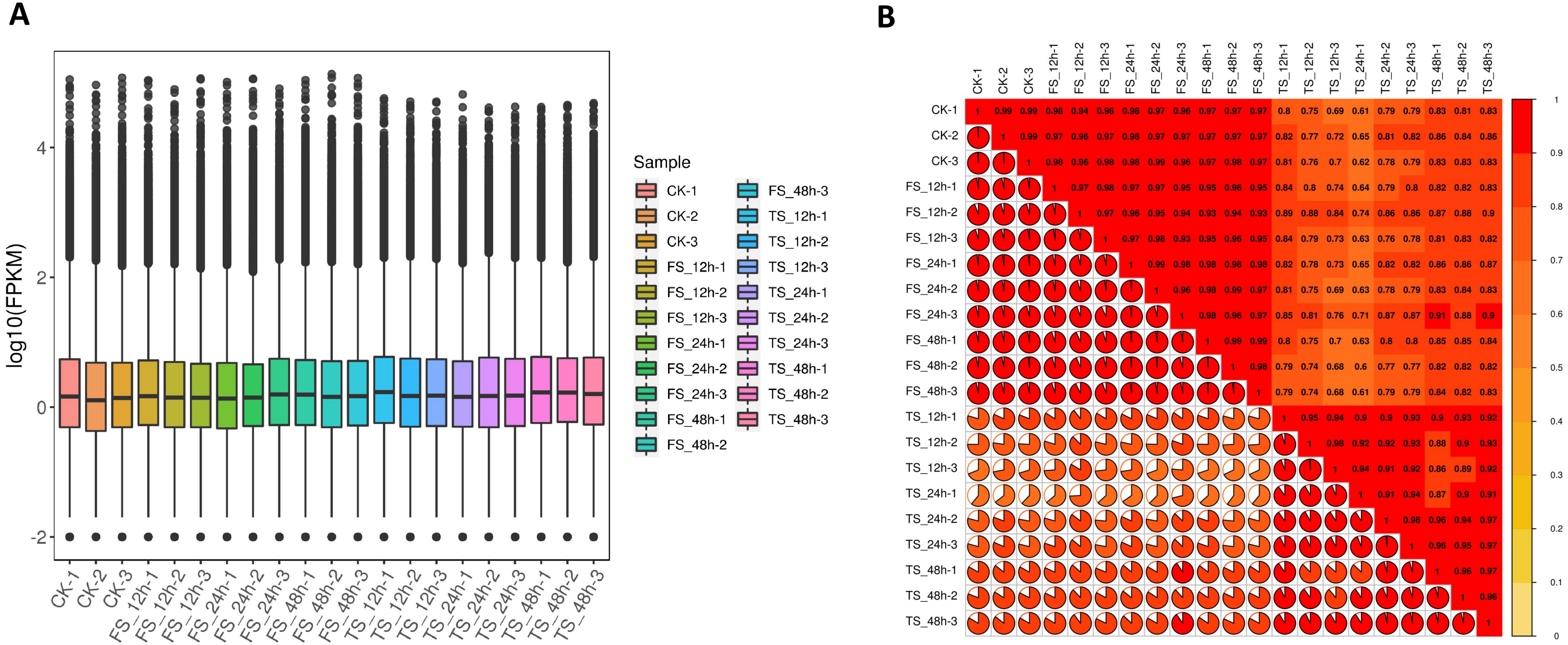

Supplement: Supplemental Information 4 — (A) FPKM expression levels distribution box plot for each group. The abscissa represents different samples, the ordinate represents the logarithmic value of the sample expression FPKM. (B) Correlation heatmap of 21 samples. The numbers in the figure indicate R2. The closer R2 is to 1, the stronger the correlation between the two replicate samples. The depth of the color indicates the strength of the correlation. FS:50 mM NaCl treatments; TS:200 mM NaCl treatments. [file peerj-09-11888-s004.png]

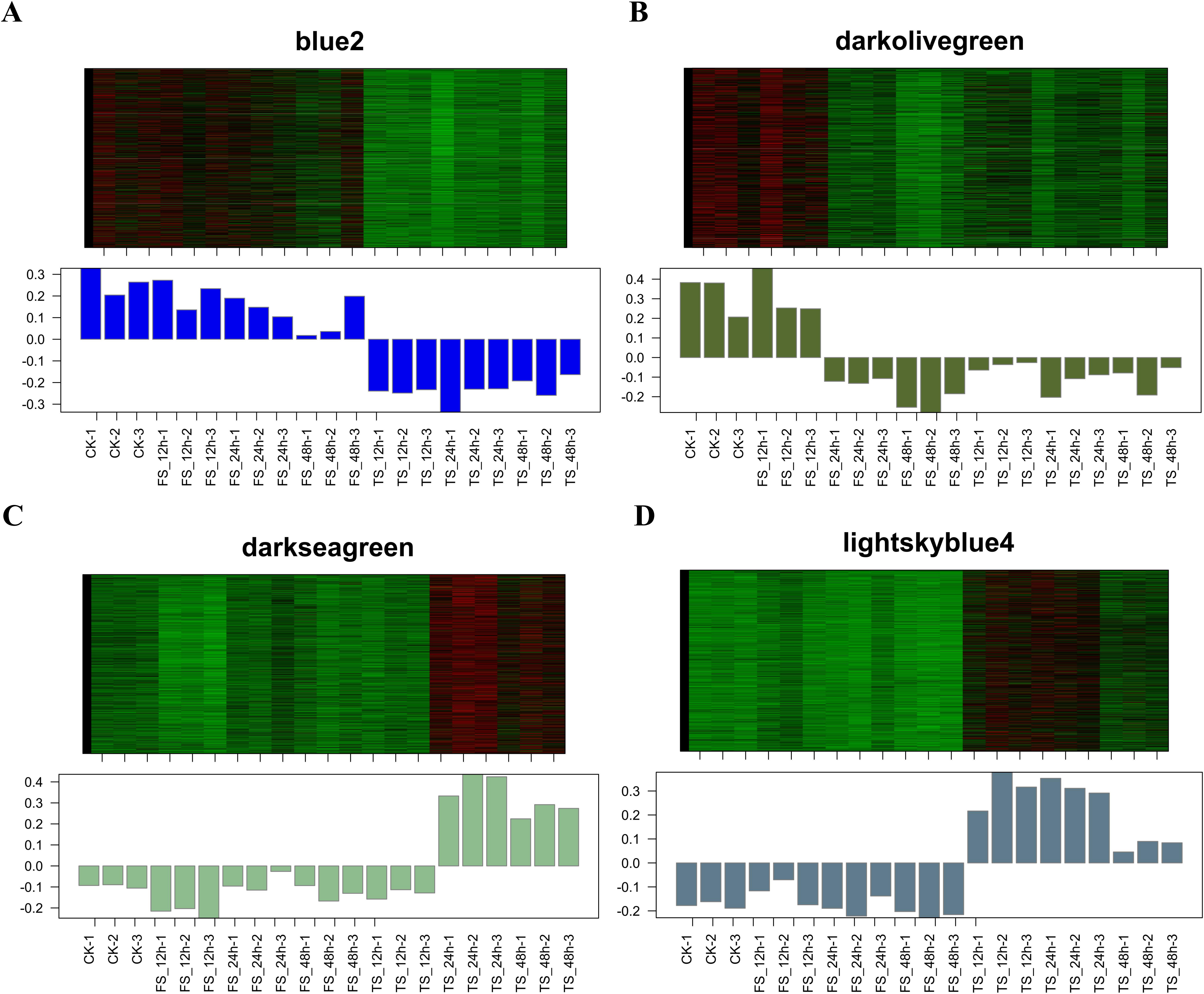

Supplement: Supplemental Information 5 [file peerj-09-11888-s005.png]

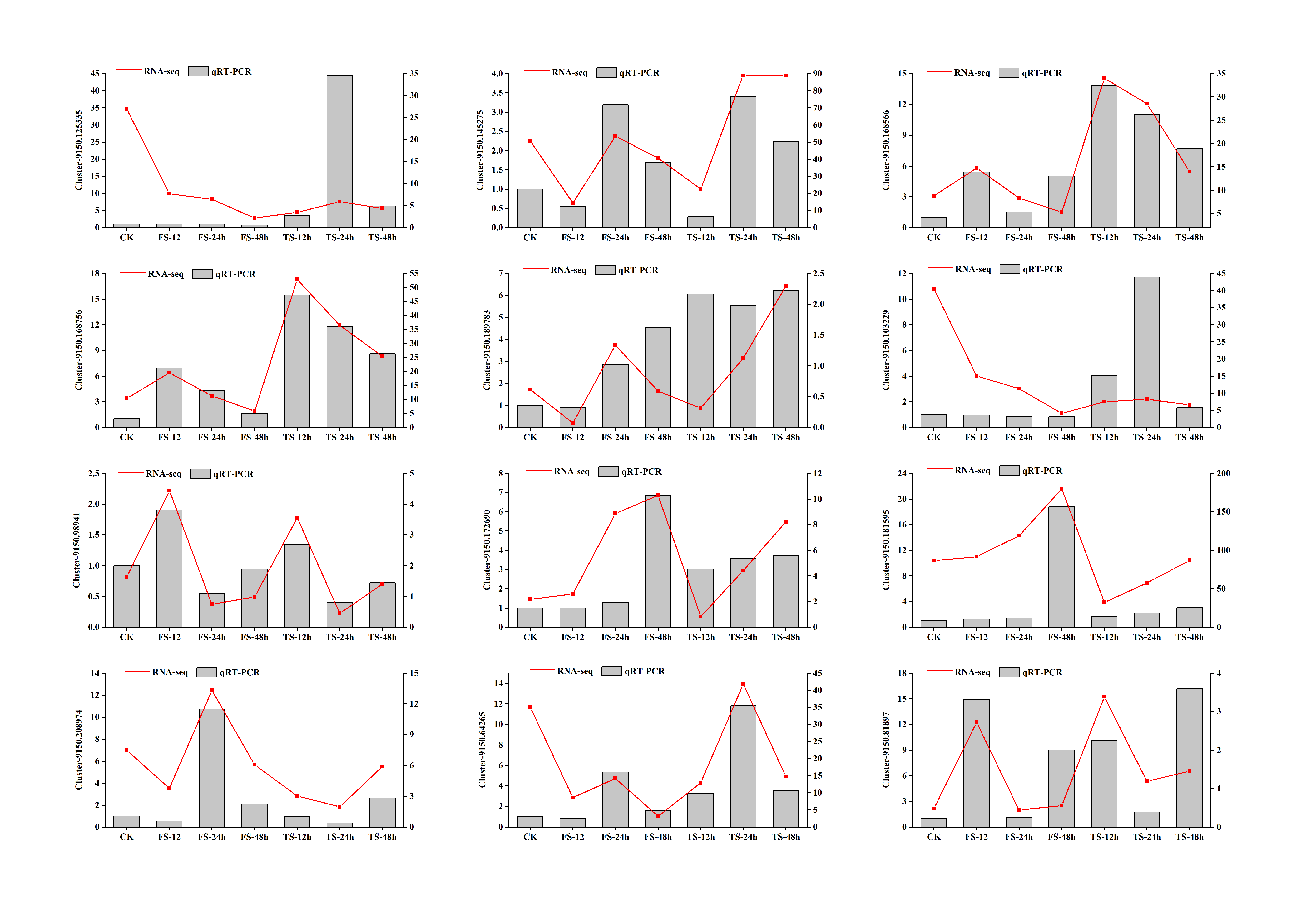

Supplement: Supplemental Information 6 — A total of 12 genes were selected for qRT-PCR analysis. The GAPDH gene was chosen as the reference gene. FS:50 mM NaCl treatments; TS:200 mM NaCl treatments. [file peerj-09-11888-s006.png]
