# Supplementary material for: Physiological changes and transcript identification in Coreopsis tinctoria Nutt. in early stages of salt stress
Source: PeerJ. 2021 Aug 9;9:e11888. doi: 10.7717/peerj.11888 (PMC8359800; doi:10.7717/peerj.11888)
Supplement: Supplemental Information 7 [file peerj-09-11888-s007.docx]

| **Table S1** Primer information | | |
| --- | --- | --- |
|  | Primer name | Primer sequence（5'-3') |
| Reference gene | sun flower(*Helianthus annuus* L)-gapdh（1）-S | CAAAGTCGTCTCTGACCGTAACC |
|  | sun flower(Helianthus annuus L)-gapdh（1）-A | CGGTGTAGGAATGAGTGGTGGT |
| 1 | Cluster-9150.81897-S | GCCAATAAAGCAACCCAACG |
|  | Cluster-9150.81897-A | GTTGAAGGTTCTTAGATGTTGGGG |
| 2 | Cluster-9150.98941-S | GAACCCGAACCATTGAAGGAG |
|  | Cluster-9150.98941-A | CGAATAACCCGTAAAAACCCC |
| 3 | Cluster-9150.103229-S | CTCGTTTCTATGGTGGAACTTGG |
|  | Cluster-9150.103229-A | GGTATGGTGGCGGAGATGTG |
| 4 | Cluster-9150.125335-S | TGATTCGCTGGAACCTAAACC |
|  | Cluster-9150.125335-A | TCAAACCATCACTTCCATCAGC |
| 5 | Cluster-9150.168566-S | GACAACAATGGCGGGTGCT |
|  | Cluster-9150.168566-A | CCGTCACCAACAAACTTTACACC |
| 6 | Cluster-9150.168756-S | GTGCTTGGGTAGATTCGTGGTG |
|  | Cluster-9150.168756-A | CAGGCTTTCTGATTGGATTTCG |
| 7 | Cluster-9150.172690-S | GCTCATTGCTATGCGTGTTTCT |
|  | Cluster-9150.172690-A | GAGATTGTGGTGGTGGGTTAGG |
| 8 | Cluster-9150.181595-S | GAACAGGATTCAGGGAGAAAGTG |
|  | Cluster-9150.181595-A | CGTCCTTTCCAGGGATTTAGAT |
| 9 | Cluster-9150.189783-S | TGGTGTTTTCACAGGCTACTCTCT |
|  | Cluster-9150.189783-A | TTCATCAAGAACAGGCAAGGC |
| 10 | Cluster-9150.208974-S | GTGATGAAGGATGCTTTGAGTAGG |
|  | Cluster-9150.208974-A | CACCATCGGACTCGGCTTCT |
| 11 | Cluster-9150.64265-S | TGGTTGCTGGGGTGCCTAT |
|  | Cluster-9150.64265-A | GCCACCGCCACCTTTAGTT |
| 12 | Cluster-9150.145275-S | CAAACCACAACCGACCAACA |
|  | Cluster-9150.145275-A | TTACACACGCACCTCCAAACAT |
