# Supplementary material for: Physiological changes and transcript identification in Coreopsis tinctoria Nutt. in early stages of salt stress
Source: PeerJ. 2021 Aug 9;9:e11888. doi: 10.7717/peerj.11888 (PMC8359800; doi:10.7717/peerj.11888)
Supplement: Supplemental Information 8 [file peerj-09-11888-s008.docx]

**Table S2** Summary of Illumina transcriptome sequencing analysis. CK: control; FS_12h, FS_24h, FS_48h: samples 12, 24, and 48 h after treatment with 50 mM NaCl; TS_12h, TS_24h, TS_48h: samples 12, 24, and 48 h after treatment with 200 mM NaCl, respectively; Q20: percentage of bases with a Phred value > 20; Q30: percentage of bases with a Phred value > 30.

| Sample | Replication | Raw Reads | Clean Reads | Error Rate(%) | Q20(%) | Q30(%) | GC Content(%) |
| --- | --- | --- | --- | --- | --- | --- | --- |
| CK | 1 | 42,486,616 | 41,152,076 | 0.03 | 97.79 | 93.52 | 45.69 |
|  | 2 | 50,805,280 | 49,057,376 | 0.03 | 97.90 | 93.75 | 45.67 |
|  | 3 | 41,630,370 | 40,125,080 | 0.03 | 97.67 | 93.19 | 45.66 |
| FS_12h | 1 | 41,729,416 | 40,341,386 | 0.03 | 97.85 | 93.64 | 45.5 |
|  | 2 | 44,239,554 | 42,061,306 | 0.03 | 97.77 | 93.43 | 45.41 |
|  | 3 | 46,680,716 | 44,279,018 | 0.03 | 97.81 | 93.57 | 45.55 |
| FS_24h | 1 | 43,062,750 | 41,198,368 | 0.03 | 97.88 | 93.70 | 45.86 |
|  | 2 | 42,764,138 | 41,063,412 | 0.03 | 97.88 | 93.70 | 45.74 |
|  | 3 | 42,542,792 | 41,430,006 | 0.03 | 97.8 | 93.51 | 45.61 |
| FS_48h | 1 | 48,598,398 | 47,123,794 | 0.03 | 97.94 | 93.85 | 45.83 |
|  | 2 | 46,529,598 | 44,991,328 | 0.03 | 97.71 | 93.33 | 46.05 |
|  | 3 | 42,895,840 | 41,439,068 | 0.03 | 97.90 | 93.77 | 45.97 |
| TS_12h | 1 | 42,092,418 | 40,663,520 | 0.03 | 97.82 | 93.56 | 45.06 |
|  | 2 | 44,126,000 | 42,447,260 | 0.03 | 97.7 | 93.32 | 45.18 |
|  | 3 | 45,941,240 | 44,322,950 | 0.03 | 97.89 | 93.70 | 45.16 |
| TS_24h | 1 | 45,450,696 | 43,825,624 | 0.03 | 97.89 | 93.70 | 45.06 |
|  | 2 | 43,524,820 | 41,857,976 | 0.03 | 97.62 | 93.29 | 45.32 |
|  | 3 | 41,707,118 | 40,316,152 | 0.03 | 97.78 | 93.48 | 45.19 |
| TS_48h | 1 | 42,303,970 | 41,058,444 | 0.03 | 97.93 | 93.79 | 45.34 |
|  | 2 | 41,859,288 | 40,428,078 | 0.03 | 97.89 | 93.71 | 45.21 |
|  | 3 | 42,287,338 | 41,035,960 | 0.03 | 97.77 | 93.44 | 45.27 |
