# Supplementary material for: Physiological changes and transcript identification in Coreopsis tinctoria Nutt. in early stages of salt stress
Source: PeerJ. 2021 Aug 9;9:e11888. doi: 10.7717/peerj.11888 (PMC8359800; doi:10.7717/peerj.11888)
Supplement: Supplemental Information 9 [file peerj-09-11888-s009.docx]

**Table S3** Summary of the sequence assembly results. Number: the number of sequences. Mean Length: the average length of the sequence. N50/N90: The spliced transcripts were sorted by length from long to short, and the length of the transcript that was added to the spliced transcript was not less than 50%/90% of the total length. Total bases: the total number of bases in the sequence.

| Type | Number | Mean Length | N50 | N90 | Total Bases |
| --- | --- | --- | --- | --- | --- |
| Transcript | 507230 | 678 | 864 | 325 | 344050046 |
| Unigene | 428638 | 750 | 922 | 381 | 321620652 |
