# Supplementary material for: Physiological changes and transcript identification in Coreopsis tinctoria Nutt. in early stages of salt stress
Source: PeerJ. 2021 Aug 9;9:e11888. doi: 10.7717/peerj.11888 (PMC8359800; doi:10.7717/peerj.11888)
Supplement: Supplemental Information 10 [file peerj-09-11888-s010.docx]

**Table S4** Summary of functional annotation for assembled unigenes.

| Database | Number of Genes | Percentage (%) |
| --- | --- | --- |
| NR | 270,860 | 63.19 |
| Trembl | 268,369 | 62.61 |
| GO | 227,019 | 52.96 |
| KEGG | 203,045 | 47.37 |
| SwissProt | 179,823 | 41.95 |
| Pfam | 167,403 | 39.05 |
| KOG | 146,589 | 34.20 |
| Annotated in at least one Database | 273,318 | 63.76 |
| Total Unigenes | 428,638 | 100 |
