# Supplementary material for: Physiological changes and transcript identification in Coreopsis tinctoria Nutt. in early stages of salt stress
Source: PeerJ. 2021 Aug 9;9:e11888. doi: 10.7717/peerj.11888 (PMC8359800; doi:10.7717/peerj.11888)
Supplement: Supplemental Information 11 [file peerj-09-11888-s011.docx]

| **Table S4** The first 10 GO terms with significant enrichment of blue2 and Lightskyblue4 modules | | | | |
| --- | --- | --- | --- | --- |
| Ontology | ID | Description | P-value | Count |
| Blue2 | | | | |
| Biological process | GO:0019253 | reductive pentose-phosphate cycle | 6.60E-51 | 62 |
| Biological process | GO:0019685 | photosynthesis, dark reaction | 7.82E-51 | 62 |
| Cellular component | GO:0010319 | stromule | 1.73E-21 | 32 |
| Molecular function | GO:0016984 | ribulose-bisphosphate carboxylase activity | 5.42E-19 | 18 |
| Molecular function | GO:0008974 | phosphoribulokinase activity | 8.72E-17 | 13 |
| Cellular component | GO:0010598 | NAD(P)H dehydrogenase complex (plastoquinone) | 5.54E-16 | 17 |
| Biological process | GO:0009767 | photosynthetic electron transport chain | 5.92E-14 | 29 |
| Molecular function | GO:0097718 | disordered domain specific binding | 1.86E-13 | 9 |
| Biological process | GO:0009773 | photosynthetic electron transport in photosystem I | 6.60E-13 | 17 |
| Cellular component | GO:0009295 | nucleoid | 8.47E-13 | 25 |
| Lightskyblue4 module | | | | |
| Biological process | GO:0006561 | proline biosynthetic process | 3.26E-20 | 32 |
| Biological process | GO:0055129 | L-proline biosynthetic process | 3.89E-20 | 29 |
| Molecular function | GO:0008692 | 3-hydroxybutyryl-CoA epimerase activity | 1.72E-19 | 27 |
| Molecular function | GO:0016856 | racemase and epimerase activity, acting on hydroxy acids and derivatives | 1.72E-19 | 27 |
| Molecular function | GO:0016863 | intramolecular oxidoreductase activity, transposing C=C bonds | 3.28E-19 | 31 |
| Molecular function | GO:0004165 | dodecenoyl-CoA delta-isomerase activity | 7.32E-19 | 28 |
| Molecular function | GO:0004300 | enoyl-CoA hydratase activity | 2.82E-18 | 27 |
| Molecular function | GO:0003857 | 3-hydroxyacyl-CoA dehydrogenase activity | 4.35E-18 | 27 |
| Biological process | GO:0006560 | proline metabolic process | 6.24E-18 | 34 |
| Molecular function | GO:0004349 | glutamate 5-kinase activity | 1.06E-17 | 25 |
