# Supplementary material for: Physiological changes and transcript identification in Coreopsis tinctoria Nutt. in early stages of salt stress
Source: PeerJ. 2021 Aug 9;9:e11888. doi: 10.7717/peerj.11888 (PMC8359800; doi:10.7717/peerj.11888)
Supplement: Supplemental Information 12 [file peerj-09-11888-s012.docx]

**Table S6** Description of 25 genes in the blue2 module

| Gene ID | Gene name | Description |
| --- | --- | --- |
| Cluster-9150.174633 | RuBPcase | ribulose bisphosphate carboxylase small chain |
| Cluster-9150.174634 |  |  |
| Cluster-9150.174640 |  |  |
| Cluster-9150.175655 |  |  |
| Cluster-9150.175657 |  |  |
| Cluster-9150.177631 |  |  |
| Cluster-9150.177636 |  |  |
| Cluster-9150.177639 |  |  |
| Cluster-9150.178675 |  |  |
| Cluster-9150.170336 | RpiA | ribose 5-phosphate isomerase A |
| Cluster-9150.175203 | GS | glutamine synthetase |
| Cluster-9150.176834 | CHY | 3-hydroxyisobutyryl-CoA hydrolase |
| Cluster-9150.181014 | GAPD | glyceraldehyde-3-phosphate dehydrogenase (NADP+) (phosphorylating) |
| Cluster-9150.281862 | MDH | malate dehydrogenase (oxaloacetate-decarboxylating)(NADP+) |
| Cluster-9150.190023 | [EC:3.4.19.12] | OTU domain-containing protein 6 |
| Cluster-9150.135571 | others | solute carrier family 35, member E1 |
| Cluster-9150.177438 |  |  |
| Cluster-9150.158304 |  | glycine cleavage system H protein |
| Cluster-9150.182099 |  |  |
| Cluster-9150.156503 |  | light-harvesting complex I chlorophyll a/b binding protein 2 |
| Cluster-9150.162845 | unknown | / |
| Cluster-9150.170813 |  |  |
| Cluster-9150.174689 |  |  |
| Cluster-9150.176833 |  |  |
| Cluster-9150.236840 |  |  |
