# Supplementary material for: Physiological changes and transcript identification in Coreopsis tinctoria Nutt. in early stages of salt stress
Source: PeerJ. 2021 Aug 9;9:e11888. doi: 10.7717/peerj.11888 (PMC8359800; doi:10.7717/peerj.11888)
Supplement: Supplemental Information 13 [file peerj-09-11888-s013.docx]

| **Table S7** Description of 37 genes in the Lightskyblue4 module | | |
| --- | --- | --- |
| gene ID | Gene Name | Description |
| Cluster-9150.160760 | P5CS | delta-1-pyrroline-5-carboxylate synthetase |
| Cluster-9150.168929 |  |  |
| Cluster-9150.175497 |  |  |
| Cluster-9150.215535 |  |  |
| Cluster-9150.150117 | PSA | puromycin-sensitive aminopeptidase |
| Cluster-9150.184199 |  |  |
| Cluster-9150.164085 | P5CDH | 1-pyrroline-5-carboxylate dehydrogenase |
| Cluster-9150.213601 |  |  |
| Cluster-9150.201886 | PSA | puromycin-sensitive aminopeptidase |
| Cluster-9150.171835 | AASS | alpha-aminoadipic semialdehyde synthase |
| Cluster-9150.182063 |  |  |
| Cluster-9150.176006 | AGT | alanine-glyoxylate transaminase / (R)-3-amino-2-methylpropionate-pyruvate transaminase |
| Cluster-9150.188322 |  |  |
| Cluster-9150.188535 | RS | raffinose synthase |
| Cluster-9150.200200 |  |  |
| Cluster-9150.150167 | UGE | UDP-glucose 4-epimerase |
| Cluster-9150.169781 | G6PDH | glucose-6-phosphate 1-dehydrogenase |
| Cluster-9150.173822 | INF2 | formin 2 |
| Cluster-9150.180692 | PP2C | protein phosphatase 2C |
| Cluster-9150.185696 | IRAK-4 | interleukin-1 receptor-associated kinase 4 |
| Cluster-9150.218943 | E3UBPL | E3 ubiquitin-protein ligase ATL41 |
| Cluster-9150.190387 |  | solute carrier family 36 (proton-coupled amino acid transporter) |
| Cluster-9150.166717 |  |  |
| Cluster-9150.140390 |  | transmembrane anterior posterior transformation protein 1 |
| Cluster-9150.177149 |  | epidermal growth factor receptor substrate 15 |
| Cluster-9150.154007 | [EC:5.1.3.15] | glucose-6-phosphate 1-epimerase [EC:5.1.3.15] \| (RefSeq) putative glucose-6-phosphate 1-epimerase (A) |
| Cluster-9150.158854 |  | WD repeat-containing protein 26 |
| Cluster-9150.175580 |  | COMPASS component SWD3 |
| Cluster-9150.188472 |  | DnaJ homolog subfamily B member 11 |
| Cluster-9150.202948 | [EC:2.7.7.7] | DNA polymerase kappa |
| Cluster-9150.171416 | unknown | / |
| Cluster-9150.173210 |  |  |
| Cluster-9150.178124 |  |  |
| Cluster-9150.184409 |  |  |
| Cluster-9150.189141 |  |  |
| Cluster-9150.191042 |  |  |
| Cluster-9150.216387 |  |  |
